# Structure and Mechanism of Glycine Receptor Elucidated by Cryo-Electron Microscopy

**DOI:** 10.3389/fphar.2022.925116

**Published:** 2022-08-09

**Authors:** Hongtao Zhu

**Affiliations:** Laboratory of Soft Matter Physics, Institute of Physics, Chinese Academy of Sciences, Beijing, China

**Keywords:** glycine receptor, cryo-EM, agonist, inhibitory receptor, antagonist, partial agonist, potentiator, gating mechanism

## Abstract

Glycine receptors (GlyRs) are pentameric ion channels that mediate fast inhibitory neurotransmission. GlyRs are found in the central nervous system including the spinal cord, brain stem, and cerebellum, as well as in the retina, sperm, macrophages, hippocampus, cochlea, and liver. Due to their crucial roles in counter-balancing excitatory signals and pain signal transmission, GlyR dysfunction can lead to severe diseases, and as a result, compounds that modify GlyR activity may have tremendous therapeutic potential. Despite this potential, the development of GlyR-specific small-molecule ligands is lacking. Over the past few years, high-resolution structures of both homomeric and heteromeric GlyRs structures in various conformations have provided unprecedented details defining the pharmacology of ligand binding, subunit composition, and mechanisms of channel gating. These high-quality structures will undoubtedly help with the development of GlyR-targeted therapies.

## Introduction

Glycine receptors (GlyRs) are ligand-gated ion channel that are members of the Cys-loop superfamily which also includes the GABA_A_ receptor (GABA_A_R), nicotinic acetylcholine receptor (nAChR), serotonin type-3 receptor, and zinc-activated ion channel (Lester et al., 2004). GlyRs can be activated by a variety of endogenous ligands including the full agonist glycine as well as the partial agonists taurine, β-alanine, and GABA (Lynch, 2004). The activation of GlyRs caused by agonist binding results in a Cl^−^ flow across the membrane that is regulated by the Cl^−^ equilibrium potential and induces the membrane hyperpolarization, which in turn inhibits neuronal activity (Legendre, 2001; Lynch, 2004).

There are four known GlyR α subunits (α1–α4) and one β subunit identified by molecular cloning (Grenningloh et al., 1987; Grenningloh et al., 1990; Akagi et al., 1991; Kuhse et al., 1991; Matzenbach et al., 1994). Each GlyR subunit is composed of an extracellular domain (ECD), a transmembrane domain (TMD), and a long intracellular loop connecting transmembrane domains M3 and M4. The binding pockets are formed by two adjacent subunits located in the ECD (Lynch, 2009). All of the GlyR α subunits have substantial sequence similarity, exceeding 90%, whereas the GlyR β subunit has a considerable sequence variation when compared with GlyR α subunits (Lynch, 2009). Functional GlyRs include homomeric α GlyRs and heteromeric α-β GlyRs. In the prenatal stage, the predominant type of GlyR is homomeric α2, whereas the adult GlyR types are mainly heteromeric α1-β GlyRs (Becker et al., 1988; Lynch, 2009). The GlyR subunit α3 is involved in nociceptive signaling pathways and function as a novel analgesic candidate (Huang et al., 2017b; Hussein et al., 2019; Zeilhofer et al., 2021). In 1982, GlyRs were isolated for the first time *via* strychnine affinity chromatography (Pfeiffer et al., 1982). The structural investigations of GlyR, on the other hand, are behind. Significant progresses have been achieved in studying the structures of GlyR as a result of the development of cryo-EM (Du et al., 2015; Kumar et al., 2020; Yu H. et al., 2021a; Yu J. et al., 2021b; Zhu and Gouaux, 2021). At present, high resolution GlyR structures bound with different ligands have been reported (Table 1). Also, these structures have revealed crucial information about the ligand binding and gating mechanism. In this minireview, I have discussed the recent progress in elucidating the structures of GlyR–ligand complexes and progress in elaborating the gating mechanism in GlyRs using single particle cryo-electron microscopy (cryo-EM).

**TABLE 1:** Summary of GlyR structures.

PDB ID	EMDB ID	Subtype	Resolution	Ligand	State*	Method	Membrane-mimic	Reference
Homomeric GlyR								
5CFB	—	α3^$^	3.0 Å	Strychnine	Closed	X-ray	DDM	Huang et al. (2015)
5TIO	—	α3^$^	3.2 Å	Glycine; AM-3607	Desensitized	X-ray	DDM	Huang et al. (2017b)
5TIN	—	α3^$@^	2.6 Å	Glycine; AM-3607	Desensitized	X-ray	DDM	Huang et al. (2017b)
5VDH	—	α3^$^	2.8 Å	AM-3607; glycine; ivermectin	Desensitized	X-ray	DDM	Huang et al. (2017a)
5VDI	—	α3^$@^	3.1 Å	AM-3607; glycine; ivermectin	Desensitized	X-ray	DDM	Huang et al. (2017a)
3JAD	6344	α1^$^	3.9 Å	Strychnine	Closed	Cryo-EM	DDM	Du et al. (2015)
3JAF	6345	α1^$^	3.8 Å	Glycine; ivermectin	Partially-Open	Cryo-EM	DDM	Du et al. (2015)
3JAE	6346	α1^$^	3.9 Å	Glycine	Open	Cryo-EM	DDM	Du et al. (2015)
6PXD	20518	α1^$^	2.9 Å	—	Apo	Cryo-EM	DDM	Yu et al. (2021b)
7MLU	23910	α1	4.1 Å	Glycine	Desensitized	Cryo-EM	DDM	Zhu and Gouaux, (2021)
7MLV^&^	23911^&^	—	3.8 Å	—	—	Cryo-EM	DDM	Zhu and Gouaux, (2021)
—	23912^&^	—	12.3 Å	—	—	Cryo-EM	DDM	Zhu and Gouaux, (2021)
6PLR	20373	α1	3.2 Å	Glycine	Desensitized	Cryo-EM	Nanodisc	Yu et al. (2021b)
6PLS	20374	α1	3.0 Å	Taurine	Desensitized	Cryo-EM	Nanodisc	Yu et al. (2021b)
6PLT	20375	α1	3.2 Å	Taurine	Closed	Cryo-EM	Nanodisc	Yu et al. (2021b)
6PLU	20376	α1	3.3 Å	GABA	Desensitized	Cryo-EM	Nanodisc	Yu et al. (2021b)
6PLV	20377	α1	3.3 Å	GABA	Closed	Cryo-EM	Nanodisc	Yu et al. (2021b)
6UBS	20714	α1	3.3 Å	—	Apo	Cryo-EM	Nanodisc	Kumar et al. (2020)
6UBT	20715	α1	3.4 Å	Glycine	Desensitized	Cryo-EM	Nanodisc	Kumar et al. (2020)
6UD3	20731	α1	3.5 Å	Glycine; picrotoxin	Open	Cryo-EM	Nanodisc	Kumar et al. (2020)
6VM0	21234	α1	3.1 Å	Glycine; ivermectin	Partially-Open	Cryo-EM	Nanodisc	Kumar et al. (2020)
6VM2	21236	α1	3.3 Å	Glycine; ivermectin	Partially-Open	Cryo-EM	Nanodisc	Kumar et al. (2020)
6VM3	21237	α1	3.0 Å	Glycine; ivermectin	Partially-Open	Cryo-EM	Nanodisc	Kumar et al. (2020)
6PM5	20388	α1	3.2 Å	Glycine	Desensitized	Cryo-EM	SMA	Yu et al. (2021b)
6PM6	20389	α1	3.2 Å	Glycine	Open	Cryo-EM	SMA	Yu et al. (2021b)
6PM4	20386	α1	3.2 Å	Glycine	Expanded-Open	Cryo-EM	SMA	Yu et al. (2021b)
6PM1	20383	α1	3.0 Å	Taurine	Desensitized	Cryo-EM	SMA	Yu et al. (2021b)
6PM2	20384	α1	3.2 Å	Taurine	Open	Cryo-EM	SMA	Yu et al. (2021b)
6PM0	20382	α1	3.1 Å	Taurine	Expanded-Open	Cryo-EM	SMA	Yu et al. (2021b)
6PM3	20385	α1	3.2 Å	Taurine	Closed	Cryo-EM	SMA	Yu et al. (2021b)
6PLX	20379	α1	2.9 Å	GABA	Desensitized	Cryo-EM	SMA	Yu et al. (2021b)
6PLY	20380	α1	2.9 Å	GABA	Open	Cryo-EM	SMA	Yu et al. (2021b)
6PLW	20378	α1	3.0 Å	GABA	Expanded-Open	Cryo-EM	SMA	Yu et al. (2021b)
6PLZ	20381	α1	3.0 Å	GABA	Closed	Cryo-EM	SMA	Yu et al. (2021b)
6PLO	20370	α1^%^	3.3 Å	GABA	Open	Cryo-EM	SMA	Yu et al. (2021b)
6PLQ	20372	α1^%^	3.4 Å	GABA	Expanded-Open	Cryo-EM	SMA	Yu et al. (2021b)
6PLP	20371	α1^%^	3.3 Å	GABA	Desensitized	Cryo-EM	SMA	Yu et al. (2021b)
Heteromeric GlyR								
7MLY	23913	α1-β	2.7 Å	Glycine	Desensitized	Cryo-EM	DDM	Zhu and Gouaux, (2021)
7KUY	23041	α2-β^$^	3.6 Å	Strychnine	Closed	Cryo-EM	Nanodisc	Yu et al. (2021a)
7L31	23048	α2-β^$^	3.8 Å	Strychnine	Closed	Cryo-EM	Nanodisc	Yu et al. (2021a)
5BKG	9404	α2-β^$^	3.8 Å	Glycine	Semi-Open	Cryo-EM	Nanodisc	Yu et al. (2021a)
5BKF	9403	α2-β^$^	3.6 Å	Glycine	Desensitized	Cryo-EM	Nanodisc	Yu et al. (2021a)

^∗^State claimed in the literature.

^$^The M3M4 Loop is truncated.

^@^Carrying the mutation N38Q.

^%^Carrying the mutation YGF.

^&^Partially assembled homomeric GlyR.

### Full Agonist: Glycine

**FIGURE 1:**
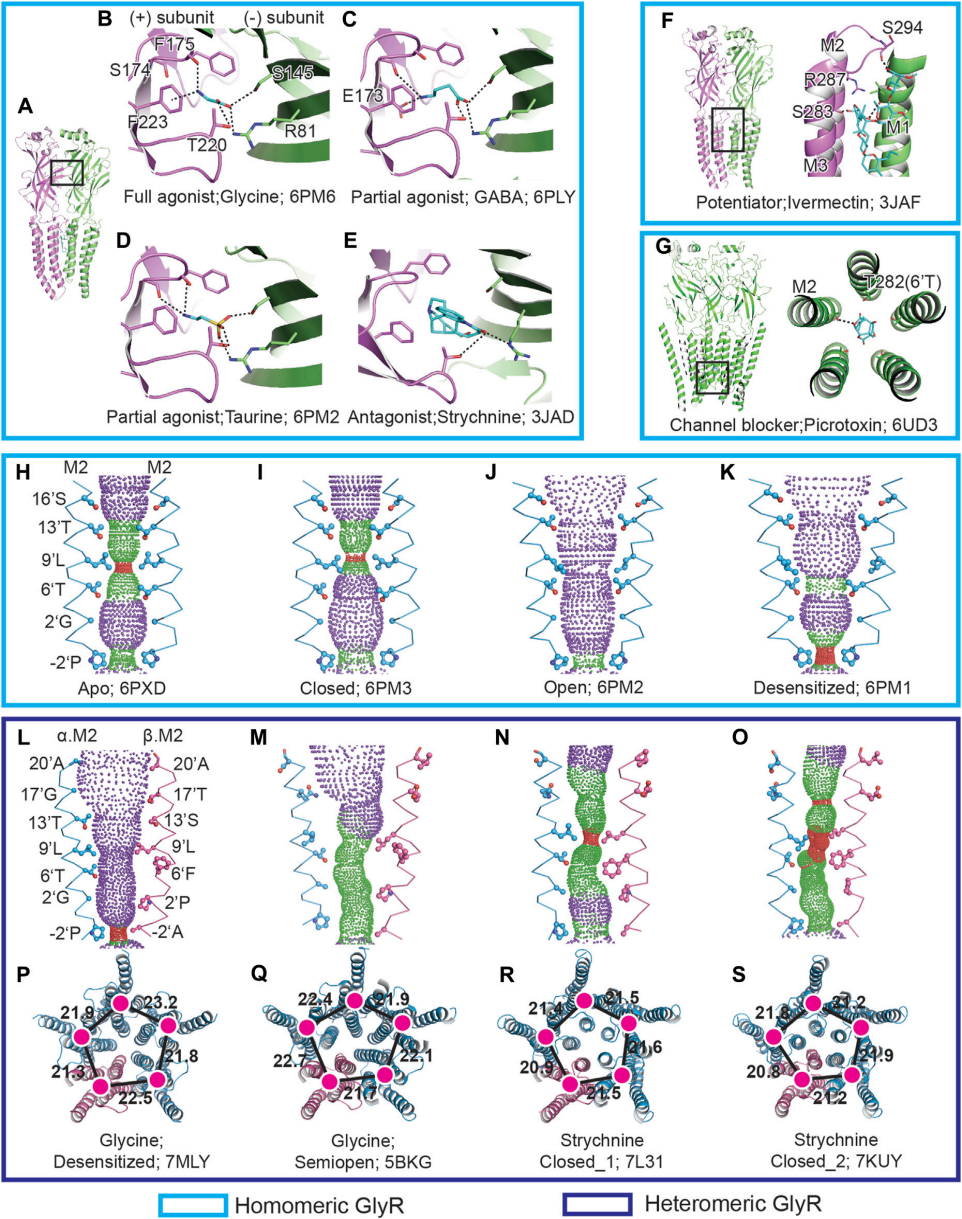
GlyR–ligand interactions and the ion channel permeation pathway. **(A)**, Side view of isolated homomeric GlyR dimer in cartoon representation. The principle (+) and complementary (−) subunit are colored in pink and green, respectively. The boxed area is enlarged in **(B–E)**. **(B–E)** Views of binding pockets of homomeric GlyR bound with glycine **(B)**, GABA **(C)**, taurine **(D)**, and strychnine **(E)**. The ligand molecules are shown in stick representations with oxygen in red, nitrogen in blue, and carbon in cyan. The possible hydrogen bonds are shown as dashed lines. **(F,G)** Views of ivermectin **(F)** and picrotoxin **(G)** binding to homomeric GlyR. **(H–K)** Shape and size of the homomeric GlyR ion permeation pathway for apo **(H)**, closed **(I)**, open **(J)**, and desensitized **(K)** state. M2 helices are shown as cartoons and the side chains of pore-lining residues are in ball and stick representation. Purple, green, and red spheres define radii of >3.3 Å, 1.8–3.3 Å and <1.8 Å, respectively. **(L–O)** Shape and size of the heteromeric GlyR ion permeation pathway for desensitized **(L)**, semi-open **(M)**, strychnine-bound closed state 1 **(N)** and strychnine-bound closed state 1 **(O)**. The M2 helices from the α and β subunits are colored in blue and pink, respectively. **(P–S)** TMD of heteromeric pentamer shown in cartoon representation corresponding to **(L)** to **(O)**. The α and β subunits are colored in blue and pink, respectively. The centers of mass for TMD are shown in magenta. The neighboring distances of centers of mass are denoted in Å.

Glycine, the full agonist to GlyR that is co-released with GABA from presynaptic vesicles (Jonas et al., 1998), can efficiently activate GlyRs. The single channel recordings demonstrated that glycine elicits a maximum open probability (P_open_) of 0.97, much higher than other agonists (Yu J. et al., 2021b). A number of near-atomic resolution glycine-bound zebra fish homomeric GlyR α1 structures identified by cryo-EM have been reported (Du et al., 2015; Kumar et al., 2020; Yu J. et al., 2021b). The structures show that the glycine binding site is located at the subunit interface, with the carboxyl group at the entrance of the binding pockets. The amino group of glycine forms the cation–π interaction with F174 and F223 at the (+) subunit, while the carboxyl group forms several potential hydrogen bonds with the (−) subunit (Figures 1A,B).

GlyRs exhibit different conformations in different lipid mimic environments. In detergent, the truncated zebra fish GlyR α1 (GlyR_EM_) adopts an open conformation with the radius of 4.4 Å at −2′P (Du et al., 2015). However, subsequent molecular dynamics simulation discovered that GlyR_EM_ is still ion permeable when docked with the channel blocker picrotoxin, conflicting with the biochemical studies (Wang et al., 2006; Gonzalez-Gutierrez et al., 2017). The full-length GlyR in nanodisc, on the other hand, produced one desensitized state with a diameter ∼3 Å at −2′P (Kumar et al., 2020; Yu J. et al., 2021b). Interestingly, for the full-length zebra fish GlyR α1 extracted by 0.5% styrene maleic acid polymer (SMA) at 4°C for 1 h, three conformations, open, desensitized, and expanded-open states, were captured with diameters at −2′P of 5.6 Å, 3.0 Å, and 7.0 Å, respectively. For the expanded-open state in SMA, extra densities were observed at the end of M2 helices, the origin of which needs further investigation (Yu J. et al., 2021b). Given that SMA can preserve the lipid molecules surrounding the receptors, it is possible that these native lipid molecules aid the stabilization of GlyR at different physiological states.

### Partial Agonist and Gating Mechanism

Partial agonists, which have efficiency between full agonist and antagonist, are of interest for therapeutics. In the presence of a full agonist, the partial agonist will serve as an antagonist, competing with the full agonist for the same binding sites, as a result, reducing the full agonist’s effects (Calvey, 2008). Taurine and GABA are partial agonists on GlyR, featured with eliciting lower maximum P_open_ than the full agonist glycine (De Saint Jan et al., 2001). In 2021, the Sivilotti and Gouaux group performed extensive research on zebra fish GlyR α1 partial agonists combining electrophysiological and cryo-EM approaches (Yu J. et al., 2021b). The single channel recordings revealed that the maximum P_open_ for glycine, taurine, and GABA are 0.98, 0.66, and 0.39, respectively. Furthermore, in the presence of taurine and GABA, the single channel recordings feature with long-lived shut states that are not seen for glycine, implying an additional partial agonist bound closed state. For the partial agonists bound GlyR in SMA, in addition to the open, desensitized and expanded-open states captured for glycine, a partial agonist bound closed state, which is absent for the full agonist glycine, was captured. The partial agonists bound closed state exhibits parallel M2 helices and the side chain of 9′L pointing to the channel axis, creating a contrition less than 3 Å. Compared with glycine, more interactions were observed for taurine and GABA. A potential hydrogen bond was found between the carbonyl group of S174 and the amino group of taurine, as well as an interaction between E173 and GABA (Figures 1C,D). These additional interactions for GABA and taurine are possibly due to the ligand’s greater length. A comparison of the GlyR structures bound with full and partial agonists shows that the binding of partial agonists induces a lesser extended binding pocket than the full agonist. Moreover, the efficiency of the agonists has a close relationship with the volume of the ligands, with the more efficient of the ligand having a lower ligand volume.

Upon the binding of partial agonists, the gating cycle of GlyRs involves transitions of multiple states (Lape et al., 2008; Lape et al., 2012). At present, three physiological GlyR states bound with partial agonists have been captured: closed, open, and desensitized. The researchers hypothesized that the partial agonists bound closed state is a pre-open state between the apo and open state (Yu J. et al., 2021b). Based on the available structures, the partial agonist gating mechanism was established. During the transition from the apo to the closed state, after the ligand accesses the binding site, a contraction of the binding pocket occurs. But no conformational changes were observed for the TMD, with 9′L functioning as the constriction point (Figures 1H,I). From the closed to the open state, the binding pockets shrink more. The conformational changes that happen at the binding pockets travels down to the ECD-TMD interface, which further triggers the tilt of the M2 helices. The tilt of the M2 helices prompts the rotation of the side chain of 9′L and causes the channel to open (Figure 1J). After opening, the lower part of the M2 helices will undergo a further tilt, creating a constriction point at −2′P (Figure 1K) and blocking the ion permeation (Yu J. et al., 2021b).

### Antagonist: Strychnine

Strychnine, an alkaloid that can bind and antagonize GlyR, has been widely employed in radioligand binding and affinity purification experiments (Lynch, 2004, 2009; Breitinger and Breitinger, 2020; Cerdan et al., 2020). According to the human GlyR α3 X-ray crystal and GlyR_EM_ (Du et al., 2015; Huang et al., 2015; Yu H. et al., 2021b), strychnine shares the same binding pocket as glycine (Figure 1E). One state was captured for GlyR_EM_ bound with strychnine (Du et al., 2015), which features parallel M2 helices with 9′L pointing to the channel axis, resulting in a diameter of 3 Å and blocking the permeation pathway. In total, two states were obtained for human heteromeric GlyR α2-β bound with strychnine (Yu H. et al., 2021a), and both of the two states exhibit a constriction point at 9′L, but the conformation of their TMD are in markedly different (Figures 1N,O,R,S).

### Potentiator: Ivermectin

The ivermectin functions as the potentiator to GlyR, which can enhance the glycine sensitivity and increase the P_open_ (Shan et al., 2001a; Breitinger and Breitinger, 2020; Cerdan et al., 2020). There are several ivermectin-bound homomeric GlyR structures available (Table 1). The structures demonstrate that ivermectin is bound at the interface of M3 and M1 and forms a polar interaction with M2 (Figure 1F). Compared with the glycine-bound GlyR_EM_ open state (Du et al., 2015), when ivermectin binds with GlyR, the M2 helices undergo movement toward the pore lumen and contract the intracellular opening the ion channel at −2′P and enabling chloride ions to pass through. The cryo-EM structures also prove that the binding of ivermectin traps the zebra fish homomeric GlyR α1 at a partially-open state (Du et al., 2015), with the narrowest point at −2′P comparable to glycine bound open state in SMA (Yu J. et al., 2021b).

### Channel Blocker: Picrotoxin

Homomeric GlyR is more sensitive than heteromeric GlyR to the inhibition of the channel blocker picrotoxin (Pribilla et al., 1992). The picrotoxin IC_50_ values for homomeric and heteromeric GlyR are 18±1 and 259±44 μM, respectively (Shan et al., 2001b). The zebra fish homomeric GlyR α3 structure bound with picrotoxin (Kumar et al., 2020) shows that picrotoxin is nested between 2′G and 9′L and forms hydrogen bonds with 6′T (Figure 1G). The structural basis for heteromeric GlyR’s resistance to picrotoxin has been recently revealed (Zhu and Gouaux, 2021). The pig heteromeric GlyR structure demonstrates that the side chain bulk of 6′F on GlyR β subunit, the corresponding residue to 6′T in homomeric GlyR, on the one hand, provides a steric hindrance for picrotoxin accessing the binding site, while, on the other hand, it pushes the M2 helices away from each other and prevents picrotoxin binding.

### Heteromeric Glycine Receptors

The predominant type GlyR in adult is heteromeric GlyR (Becker et al., 1988; Lynch, 2009), which is composed of α and β subunits. Since the ligand-binding pockets are located at the interface of the subunits, appropriate knowledge of the subunit stoichiometry of heteromeric GlyRs is thus important to understand the molecular pharmacology. Several subunit stoichiometries have been proposed including 3α:2β, 2α:3β, and 4α:1β obtained by different methods (Langosch et al., 1988; Burzomato et al., 2003; Grudzinska et al., 2005; Durisic et al., 2012; Yang et al., 2012). Because α and β subunits show high similarity in the secondary and tertiary structures (Dutertre et al., 2012), the inconsistent results on the subunit stoichiometry reflect the difficulties in distinguishing these two subunits. The fundamental factor to solve the subunit stoichiometry problem is to precisely tag one of the subunits. In 2021, both the Gouaux group and the Wang group published the near-atomic structures of heteromeric GlyR using tissue-isolated and recombinant pig α1-β GlyR and recombinant human α2-β GlyR, respectively (Yu H. et al., 2021a; Zhu and Gouaux, 2021). A same subunit stoichiometry, which is 4α:1β, was achieved for both groups using different methods, and no other subunit composition was described. In the Gouaux group’s research, the native GlyRs were purified from pig’s spinal cord and the brain stem by strychnine affinity resin (Graham et al., 1985). A monoclonal antibody specific to GlyR α subunit was prepared to enable differentiation of α and β subunits. Data has suggested that N terminal–fused GFP is tolerated by GlyR (David-Watine et al., 1999). Interestingly, the Wang group employed an EGFP which was inserted between M3 and M4 helices to identify the GlyR β subunit. The 4α:1β stoichiometry provides important implications associated with heteromeric GlyR function and pharmacology, such as the clustering of heteromeric GlyR and drug development specific to heteromeric GlyRs. There are current two states reported for heteromeric GlyR bound with glycine: one is desensitized state and the other is semi-open (Table 1). In contrast to the semi-open state, the desensitized state shows a five-fold quasi-symmetrical TMD (Figures 1L,M,P,Q), which is similar to the homomeric desensitized state in SMA (Figures 1K,L).

### Assembly Pathway of Glycine Receptors

Members of the heteromeric Cys-loop family are composed of at least two different types of subunits. The investigation of the assembly intermediates can provide insights into the oligomerization process. Though research on the assembly process of nAChRs (Green and Claudio, 1993) and GABA_A_Rs (Klausberger et al., 2001) have been reported, little is known regarding the assembly pathway of heteromeric GlyR. Compared with nAChRs and GABA_A_Rs, the assembly process of heteromeric GlyR is comparatively simple due to the involvement of two types of subunits. By using strychnine affinity resin (Pfeiffer et al., 1982), the GlyR assembly intermediates were successfully isolated from the native materials (Zhu and Gouaux, 2021). A total of two assembly intermediates including a homomeric α tetramer and a homomeric α trimer were captured. However, the homomeric α dimer and β subunit containing assembly intermediates were missing, which might be due to non-functional binding pockets that needs further investigation. Given that GlyR assembly intermediates were captured by strychnine affinity resin, which demonstrates that the functional binding pockets are forming during receptor assembly. The findings reveal that the GlyR oligomerization occurs in steps, with one subunit added at each step. Because all of the assembly intermediates are α homomers, there will be insufficient supply of free α subunit. As a result, in the final step, the chance of homomeric α tetramer assembly with a β subunit to produce a heteromeric pentamer is larger than the likelihood of homomeric pentamer formation. The findings may be relevant for future drug development focusing on the GlyR assembly intermediates.

## Discussion

This review summarizes the recent progress of elucidating the structures of GlyR in a complex with different ligands at different conformations by single particle cryo-EM. These structures shed light on the gating mechanism and assembly pathway of GlyR and may provide important details for subsequent GlyR-specific drug design and screening of the authorized drugs. Despite this, little is known about the structural basis of GlyR clustering, GlyR–metal interaction, as well as other GlyR bound ligands (Cerdan et al., 2020), all of which are important goals in future.
